# Complement-Modulating Mechanisms of *Ginkgo* *biloba* Extract in Dry AMD: Insights From Molecular Docking and Dynamics Simulations

**DOI:** 10.1167/tvst.15.6.13

**Published:** 2026-06-08

**Authors:** Ting Huang, Dan Jiang, Dahu Wang, Zhiguo Dong, Yihong Hou, Xin Li, Xinli Deng, Xinquan Liu

**Affiliations:** 1 Department of Ophthalmology, Longhua Hospital Shanghai University of Traditional Chinese Medicine, Shanghai, China

**Keywords:** dry AMD, complement C3, complement C5, *Ginkgo biloba* extract, molecular docking, molecular dynamics simulation, translational pharmacology

## Abstract

**Purpose:**

To investigate the complement-modulating mechanisms of *Ginkgo biloba* extract (EGb 761) in dry age-related macular degeneration (dry AMD) through an integrative approach combining bioinformatics, molecular docking, and molecular dynamics (MD) simulations.

**Methods:**

Bioinformatics analysis was used to identify dry AMD-related targets, followed by molecular docking and 100-ns MD simulations to evaluate the binding stability and free energy of EGb 761 components with complement proteins C3 and C5.

**Results:**

Quercetin and ginkgolide B showed the strongest and most stable interactions with complement proteins C3 and C5. Persistent hydrogen bond networks were observed in the C3/ginkgolide B and C3- and C5-quercetin complexes. MM-Poisson–Boltzmann surface area analysis indicated that van der Waals and electrostatic interactions were the main contributors to binding, and key residues (C3: ASP1435, GLU1433, LYS1001, LYS1436; C5: ASP1457, GLU837, GLU932) were identified as critical hotspots.

**Conclusions:**

These results suggest that the major active components of *G.* *biloba* extract may act as natural complement modulators, stabilizing interactions with C3 and C5 to mitigate inflammation and oxidative injury in dry AMD.

**Translational Relevance:**

Because the blood–retinal barrier limits the efficacy of systemic drugs for retinal diseases, nanomaterial-based ocular delivery systems are gaining attention. However, identifying suitable bioactive compounds for nanocarrier encapsulation remains challenging. EGb 761, a standardized and clinically validated *G.* *biloba* extract with proven safety and efficacy in ophthalmology, represents a promising candidate. This study provides a mechanistic basis for its potential in dry AMD and offers insights for developing natural complement-targeting therapeutics.

## Introduction

Age-related macular degeneration (AMD) is one of the leading causes of irreversible visual impairment among individuals over 50 years of age.^1^ The disease primarily affects the central foveal region of the macula, leading to decreased visual acuity, metamorphopsia, and central scotoma. According to the Beckman AMD classification consensus,^2^ AMD can be categorized into early, intermediate, and late stages. The late stage includes atrophic (dry) and neovascular (wet) forms, with approximately 80% of cases classified as dry AMD.^3^ In 2020, the global prevalence of AMD was estimated at 8.69%, and the number of affected individuals is projected to rise from 196 million to 288 million by 2040,^4^ highlighting its importance as a major global public health concern.

The pathogenesis of AMD is closely associated with retinal pigment epithelium (RPE) dysfunction, alterations in Bruch's membrane, and excessive activation of the complement system.^5^^–^^7^ Genetic susceptibility, such as polymorphisms in CFH and C3, together with environmental factors, contributes to oxidative injury and lipofuscin accumulation in RPE cells. These processes trigger chronic inflammation and drusen formation, ultimately leading to photoreceptor apoptosis and choriocapillaris degeneration.^8^^–^^10^ Although anti–vascular endothelial growth factor therapy has shown remarkable efficacy in treating wet AMD, there is currently no effective treatment to halt the progression of the dry form. Antioxidants, complement inhibitors, and neuroprotective agents have therefore been proposed as potential therapeutic approaches for dry AMD.^11^

*Ginkgo biloba* extract (EGb 761) is a standardized phytopharmaceutical preparation containing 22% to 27% flavone glycosides (quercetin, kaempferol, and isorhamnetin) and 5% to 7% terpene lactones (ginkgolides A, B, C, and bilobalide).^12^ Owing to its antioxidant, anti-inflammatory, microcirculatory, and neuroprotective properties,^12^^–^^14^ EGb 761 has been widely applied in ophthalmic practice. Previous randomized controlled trials have suggested that EGb 761 may improve visual function in patients with age-related macular degeneration,^14^ but its underlying molecular mechanism remains largely unexplored.

Because dry AMD progresses slowly and requires long experimental observation periods, the development of therapeutic agents is often constrained.^15^ Molecular dynamics (MD) simulation, based on classical mechanics, enables time-resolved observation of drug–protein interactions at the atomic level and provides mechanistic evidence for drug discovery.^16^^,^^17^ Recent advances in enhanced sampling algorithms integrated with machine learning and data-sharing frameworks have markedly improved the sampling efficiency, reproducibility, and transparency of MD studies,^18^ making this approach a valuable tool for investigating drug–target interactions.

This study integrates bioinformatics analysis with Gaussian accelerated molecular dynamics^19^ simulations to investigate the binding characteristics and dynamic stability between the major active components of EGb 761 and the core targets associated with dry AMD. The goal is to elucidate the molecular mechanisms of EGb 761 and provide a theoretical foundation for its potential therapeutic application in dry AMD.

## Materials and Methods

### Target Screening and Pathway Enrichment Analysis Related to Dry AMD

The keywords “nonexudative age-related macular degeneration” and “dry age-related macular degeneration” were used to retrieve potential therapeutic targets of dry AMD from the GeneCards database (https://www.genecards.org). Duplicates obtained from both searches were merged. The identified targets were then submitted to the STRING 12.0 database (https://string-db.org),^20^ with the organism restricted to “*Homo sapiens*” and the minimum required interaction score set to “high confidence.” The resulting protein–protein interaction (PPI) network was analyzed, and key Kyoto Encyclopedia of Genes and Genomes (KEGG) signaling pathways were identified based on the enrichment results.

### Structure Preparation and Molecular Docking

The signaling pathways most closely associated with dry AMD, as identified in section 1.1, were further analyzed to extract their relevant proteins. The amino acid sequences of these core targets were obtained from the UniProt database (https://www.uniprot.org/). The sequences were then uploaded to the AlphaFold Server (https://golgi.sandbox.google.com/about) for structure prediction using AlphaFold3, a deep learning–based protein structure prediction tool that provides high-accuracy tertiary structure models.^21^ Default parameters were applied to ensure the reliability and reproducibility of the predicted structures.

To evaluate the reliability of the AlphaFold-predicted structures, the three-dimensional models of the target proteins were structurally superimposed with experimentally determined crystal structures retrieved from the Protein Data Bank (PDB, https://www.rcsb.org). Structural alignment and root mean square deviation (RMSD) calculations were performed using PyMOL 2.5 based on the Cα atoms to assess the overall conformational similarity between the predicted models and the corresponding experimental structures. Lower RMSD values indicate a higher degree of structural agreement between the two models.^22^

The three-dimensional structures of the main active compounds in EGb 761, including quercetin, kaempferol, isorhamnetin, ginkgolide A, ginkgolide B, ginkgolide C, and bilobalide, were downloaded from the PubChem database (https://pubchem.ncbi.nlm.nih.gov/).

Before docking, all solvent molecules, ions, and co-crystallized ligands were removed from the receptor proteins using PyMol 2.5. The Open Babel software (v3.0.1) was used to convert the ligand structures into the mol2 format. The processed receptor protein files were imported into AutoDock Tools (v1.5.6), polar hydrogens and Kollman charges were added, and the structures were saved as PDBQT files. Ligands were similarly prepared by adding Gasteiger charges and identifying rotatable bonds before being converted to PDBQT format. Molecular docking was performed using AutoDock Vina (v1.1.2),^23^ which employs the Broyden–Fletcher–Goldfarb–Shanno optimization algorithm to predict optimal binding conformations of ligands within receptor active sites. All torsional bonds of the ligands were allowed to rotate during docking. Ligand–protein complexes with binding affinities lower than –6 kcal/mol were considered to have favorable binding interactions and were subsequently used for molecular dynamics simulations. The docking poses were visualized using PyMol to display the spatial interactions between ligands and receptors.

### Molecular Dynamics Simulation

Based on the molecular docking results, MD simulations were performed under periodic boundary conditions using the Groningen Machine for Chemical Simulations (GROMACS, v5.0) software package with the CHARMM36 force field on a high-performance computing cluster.^24^ MD simulations rely on Newtonian mechanics to describe the motion of molecular systems, allowing for the sampling of ensembles composed of different molecular states and for the calculation of macroscopic thermodynamic properties to evaluate complex stability. To improve computational efficiency, only the protein regions in close proximity to the ligand binding sites were retained for system construction. Ligand topology files were generated using the CHARMM General Force Field (CGenFF).^25^ The CHARMM-modified TIP3P water model was used to solvate the system, and potassium and chloride ions were added to achieve electrostatic neutrality. The entire system underwent energy minimization using the steepest descent algorithm to remove unfavorable steric interactions. After energy minimization, equilibration was conducted in two phases: the NVT ensemble (constant number of particles, volume, and temperature) for 100 ps and the NPT ensemble (constant number of particles, pressure, and temperature) for 500 ps. The temperature was maintained at 300 K using the V-rescale thermostat, and the pressure was controlled at 1 bar with the Parrinello–Rahman barostat. Following equilibration, a 100-ns production simulation was performed at 300 K with a time step of 2.0 fs. Trajectory coordinates were saved every 1 ps for subsequent analyses.

### Molecular Dynamics Simulation Analysis

After the completion of the simulations, detailed structural and dynamic analyses were performed. The overall stability of the protein–ligand complexes was evaluated by calculating the RMSD, while the flexibility of individual amino acid residues was assessed through the root mean square fluctuation (RMSF). The radius of gyration (Rg) was computed to examine the compactness of the protein structure, and the number of hydrogen bonds was analyzed to better characterize intermolecular interactions during the simulation. To further quantify the binding affinity between the ligand and the target protein, the molecular mechanics–Poisson–Boltzmann surface area (MM-PBSA) method was applied to the last 20 ns of the MD trajectories. The gmx_MMPBSA tool^26^ was used to perform all MM-PBSA calculations:

$$\begin{array}{c} \Delta G_{bind}=G_{COM}-G_{REC}-G_{LIG} \end{array}$$

In this equation, Δ*G_bind_* represents the binding free energy, and each term on the right-hand side is defined as

$$\begin{array}{c} G_{x}=E_{MM}+G_{sol}-TS \end{array}$$

In the above equation, *E_MM_* and *G_sol_* represent the molecular mechanical energy in the gas phase and the solvation free energy, respectively, while *TS* denotes the conformational entropy. Therefore, the Δ*G_bind_* can be further expressed as

$$\begin{array}{c} \Delta G_{bind}=\Delta E_{MM}+\Delta G_{sol}-T\Delta S \end{array}$$

The sum of Δ*E_MM_*​ and Δ*G_sol_* corresponds to the enthalpic contribution to ligand–protein binding, where Δ*E_MM_*​ is further decomposed into the sum of bonded and nonbonded interaction energy terms as

$$\begin{array}{ccc} & & \;\Delta E_{MM}=\\ & & \;\left(\Delta E_{bond}+\Delta E_{angle}+\Delta E_{dihedral}\right)+\left(\Delta E_{ele}+AE_{vdw}\right) \end{array}$$

Δ*E_ele_*​ and Δ*E_vdw_* represent the electrostatic and van der Waals interaction energy changes, respectively. Because no covalent bonds were formed between the ligand and the protein during the simulations, the bonded energy contribution was considered zero. In addition, the solvation free energy was composed of polar and nonpolar components as

$$\begin{array}{c} \Delta G_{sol}=\Delta G_{PB}+\Delta G_{nonpolar} \end{array}$$

Δ*G_PB_​* represents the change in polar solvation free energy, while Δ*G_nonpolar_* is proportional to the variation in solvent-accessible surface area (Δ*SASA*).

Finally, based on the MM-PBSA binding free energy results, per-residue free energy decomposition was performed within a 5 Å radius around each ligand to identify the key amino acid residues involved in ligand binding within the protein active site pocket.

## Results

### Gene Ontology, KEGG, and Reactome Enrichment Analyses of Genes Associated With Dry AMD

**Figure 1.**
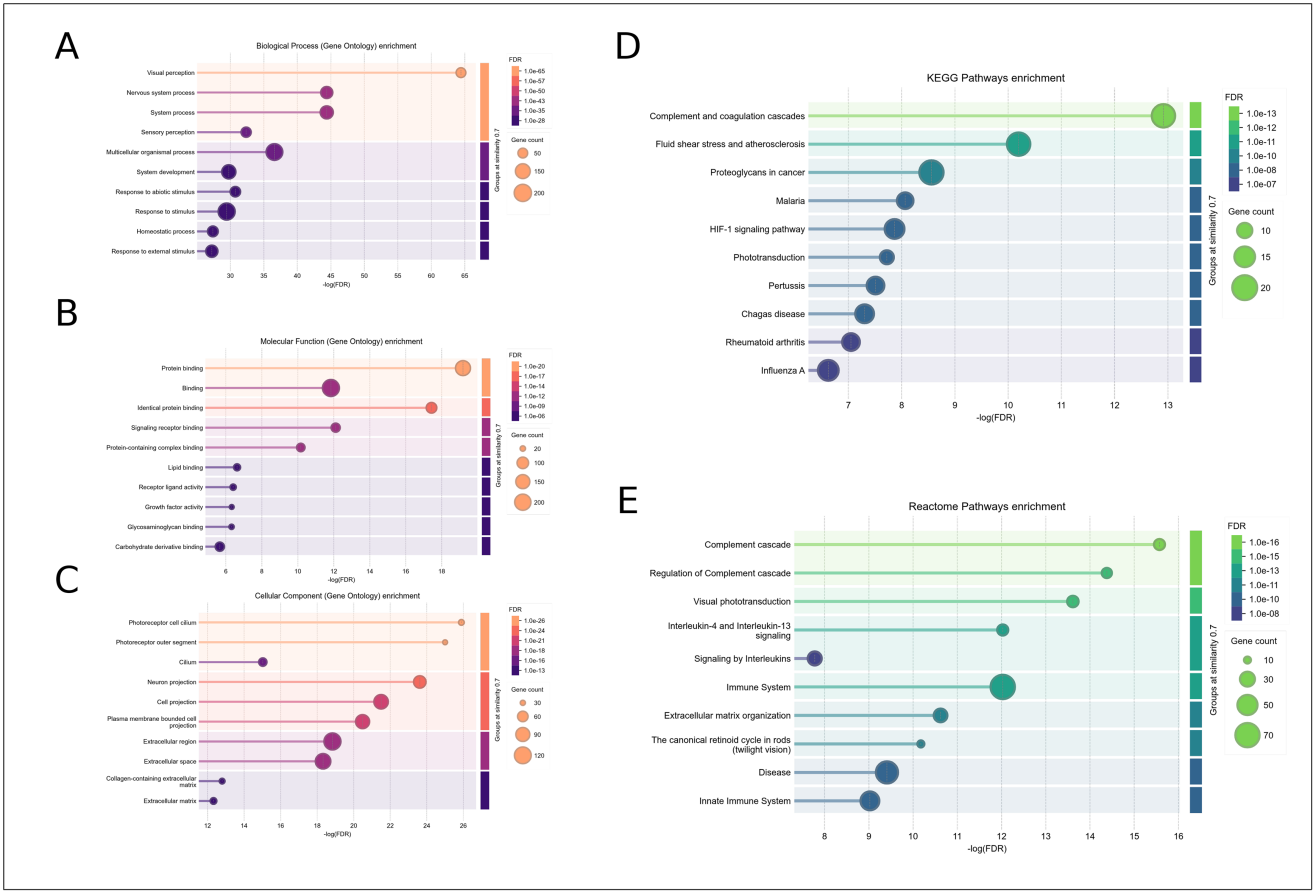
Gene Ontology (GO), KEGG, and Reactome enrichment analyses of genes associated with dry AMD. (**A**) Biological process (BP), (B) molecular function (MF), and (C) cellular component (CC). (**A**–**C**) GO enrichment analyses at three levels. The top enriched terms were mainly related to visual perception, nervous system process, sensory perception, photoreceptor cell structure, and protein binding. (**D**) KEGG pathway enrichment analysis showing significant involvement of the genes in complement and coagulation cascades, fluid shear stress and atherosclerosis, HIF-1 signaling, and phototransduction pathways. (**E**) Reactome pathway enrichment analysis indicating strong enrichment in complement activation, regulation of the complement cascade, interleukin signaling, immune system response, extracellular matrix organization, and visual phototransduction. Circle size represents gene count; color intensity reflects adjusted false discovery rate values.

Comprehensive Gene Ontology and pathway enrichment analyses were performed for the target genes associated with dry AMD. The results demonstrated significant enrichment of these genes across multiple biological levels (Figs. 1A–C).

At the biological process level, the differentially expressed genes were predominantly enriched in visual perception, nervous system process, sensory perception, system development, and response to stimulus (Fig. 1A). At the cellular component level, the enriched genes were mainly localized to the photoreceptor cell cilium, photoreceptor outer segment, neuron projection, and collagen-containing extracellular matrix (Fig. 1B). At the molecular function level, the genes were significantly enriched in protein binding, identical protein binding, signaling receptor binding, and receptor ligand activity (Fig. 1C).

Further KEGG and Reactome pathway enrichment analyses (Figs. 1D, 1E) revealed that the AMD-related genes were mainly involved in complement and coagulation cascades, fluid shear stress and atherosclerosis, HIF-1 signaling, phototransduction, and immune-related pathways. Among these, the complement and coagulation cascade pathway showed the highest enrichment, highlighting the central role of complement activation in the pathogenesis of dry AMD (Fig. 1D). The Reactome analysis^27^ (Fig. 1E) further confirmed these findings, demonstrating significant enrichment in the complement cascade and its regulation, interleukin signaling, immune system response, extracellular matrix organization, and visual phototransduction.

**Figure 2.**
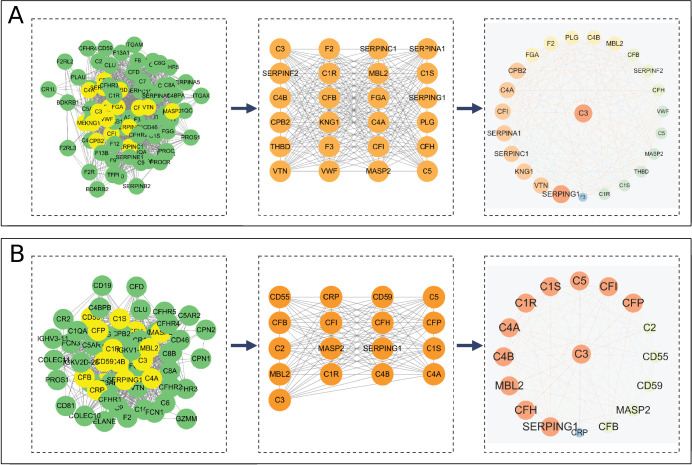
PPI network construction and identification of core genes based on KEGG and Reactome pathways. (**A**) KEGG-related PPI network. (**B**) Reactome-based PPI network.

To identify the key regulatory proteins involved in the pathogenesis of dry AMD, PPI networks were constructed based on the KEGG and Reactome results (Figs. 2A, 2B). The interaction data were obtained from the STRING database and visualized using Cytoscape 3.7.2. In the KEGG-related network (Fig. 2A), proteins exhibited dense interconnections, and MCODE clustering analysis identified C3, C4A, C4B, C5, C9, and CFH as hub genes. Similarly, in the Reactome network (Fig. 2B), the core genes included CFB, CFH, C2, C4B, C5, C7, and C9. The results from both analyses were highly consistent, indicating that the complement activation pathway represents the central regulatory module in the molecular network of dry AMD.

Taken together, the enrichment and interaction analyses suggest that C3, CFH, C5, and C9 may serve as key molecular targets through which EGb 761 exerts its protective effects on dry AMD. Based on literature evidence^6^^,^^8^ and biological relevance, C3 and C5 were selected for subsequent molecular docking and MD simulation studies.

### Validation of AlphaFold-Predicted Protein Structures

As shown in Figure 4A, the AlphaFold-predicted structures of C3 and C5 exhibited good overall structural agreement with the experimentally determined crystal structures retrieved from the PDB (C3: PDB ID 2I07; C5: PDB ID 3CU7). Structural superposition analysis revealed that the RMSD between the predicted C3 model and the corresponding crystal structure was 2.122 Å, while that for C5 was 1.325 Å. These findings indicate that the AlphaFold-predicted models possess satisfactory structural reliability, supporting their use in subsequent molecular docking and molecular dynamics simulations (Fig. 4A).

### Molecular Docking

**Figure 3.**
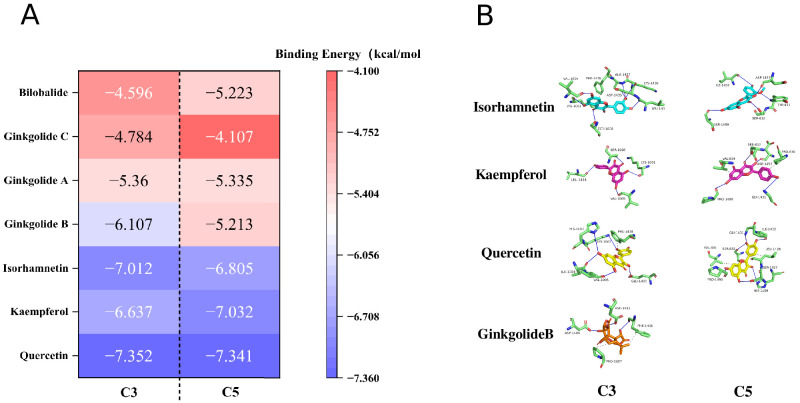
Molecular docking analysis of the major active components of EGb 761 with complement proteins C3 and C5. (**A**) Heatmap of docking scores. (**B**) Representative docking conformations of the top-scoring ligand–protein complexes.

Based on the criterion that ligands with docking scores greater than 6 kcal/mol exhibit good binding affinity with their target proteins, the results indicated that quercetin, kaempferol, and isorhamnetin showed strong binding potential toward both complement C3 and complement C5, whereas ginkgolide B displayed favorable affinity only with complement C3 (Fig. 3A).

Specifically, the binding interactions between these ligands and complement proteins were mainly stabilized by hydrogen bonds, hydrophobic contacts, and π–π stacking interactions. Ginkgolide B interacted with C3 through hydrogen bonds with ASP1404, ASP1435, and PHE1438 and formed hydrophobic contacts with PRO1007 and PHE1438. Isorhamnetin established hydrogen bonds with SER1008, LYS1001, VAL1005, ASP1435, ALA1437, LYS1436, and LEU1434 of C3 and hydrophobic interactions with PHE1438. Kaempferol formed hydrogen bonds with LEU1434, SER1008, LYS1001, and VAL1005 of C3. Quercetin interacted with C3 via hydrogen bonds with HIS1002, ILE1004, VAL1005, GLU1433, and LYS1001 and hydrophobic interactions with PHE1438. Regarding C5, isorhamnetin formed hydrogen bonds with SER1489, ILE1432, ASP1457, TYR831, and SER832. Kaempferol interacted with C5 through hydrogen bonds with PRO1490, GLY1431, SER832, and ASP1457, accompanied by hydrophobic contacts with VAL834. Quercetin bound to C5 through hydrogen bonds with SER832, GLY1431, ILE1432, SER1427, and HIS1459 and formed hydrophobic interactions with VAL834 (Fig. 3B).

### Molecular Dynamics Simulation

To further validate the molecular docking results, 100-ns MD simulations were performed for the protein–ligand complexes using GROMACS 5.0 with the CHARMM36 force field on a high-performance computing cluster. Multiple parameters were analyzed to evaluate the trajectories of the complexes after simulation.

### RMSD

The RMSD was calculated to evaluate the average positional changes of atoms in the system relative to the initial structure at each time point during the simulation. This parameter reflects the degree of atomic displacement over time within the system. For the protein backbone, the RMSD value indicates the point at which the protein–ligand complex reaches equilibrium.

**Figure 4.**
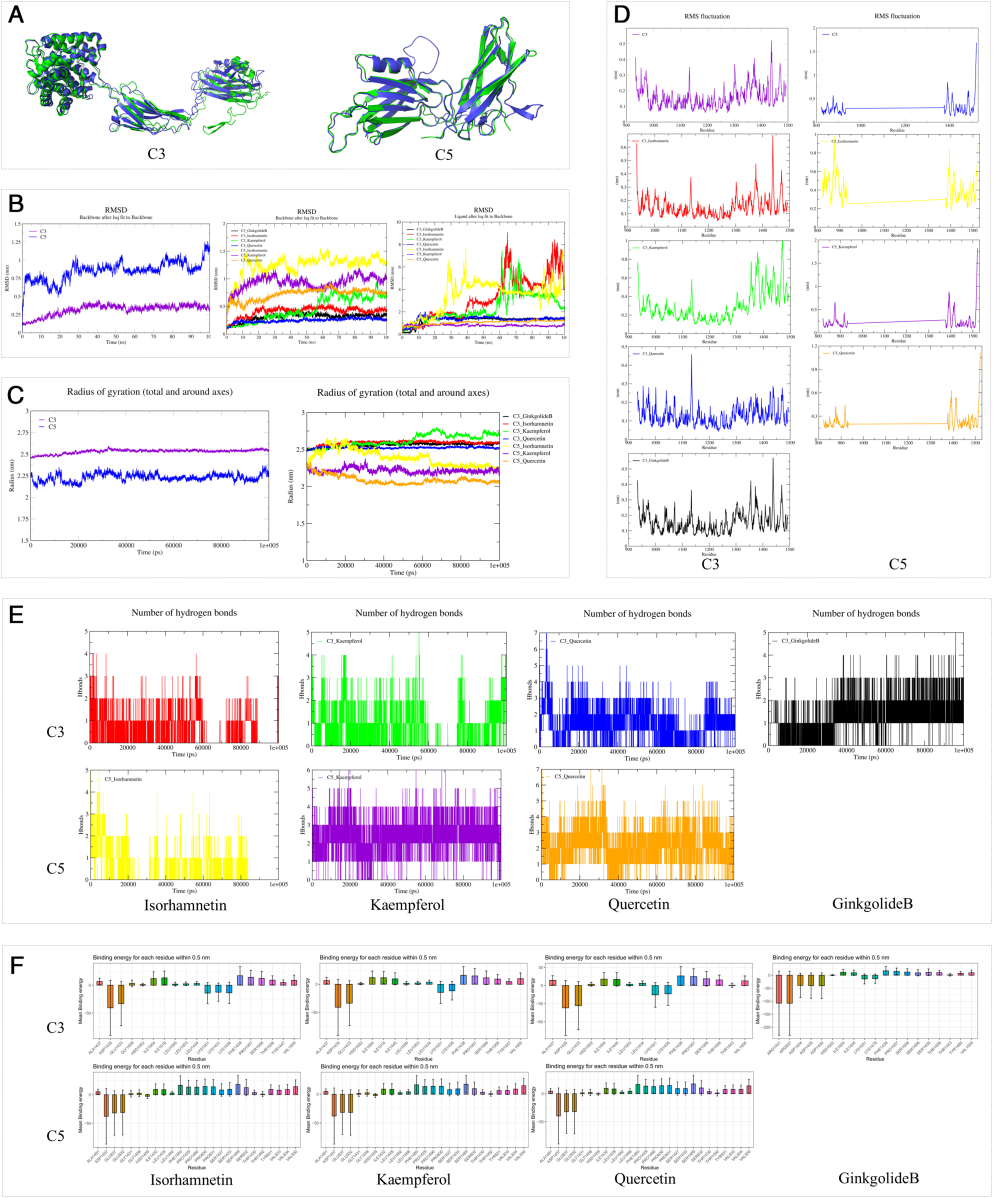
Molecular dynamics simulation analysis of EGb 761 active compounds bound to complement proteins C3 and C5. (**A**) Structural comparison of complement proteins C3 and C5. Crystal structures (C3: PDB 2I07; C5: PDB 3CU7) are shown in *green*, and AlphaFold3-predicted structures are shown in *blue*. (**B**) RMSD profiles during molecular dynamics simulations. *Left*: backbone RMSD of complement proteins C3 and C5. *Middle*: backbone RMSD of the protein–ligand complexes. *Right*: RMSD of ligands relative to the protein backbone. (**C**) Rg profiles during molecular dynamics simulations. *Left*: Rg of complement proteins C3 and C5. *Right*: Rg of the protein–ligand complexes. (**D**) RMSF profiles of complement proteins during molecular dynamics simulations. (**E**) Hydrogen bond analysis of protein–ligand complexes during molecular dynamics simulations. The number of hydrogen bonds formed between each ligand and complement proteins C3 and C5 is shown as a function of simulation time. (**F**) MM-PBSA binding free energy and per-residue energy decomposition analyses of ligand binding to complement proteins C3 and C5. The binding energy contributions of residues within 0.5 nm of the ligands are presented.

To further assess the influence of ligand binding on protein structural stability, the backbone RMSD of the apo forms of C3 and C5 was calculated as a baseline reference. The results showed that the backbone RMSD of both the apo proteins and the corresponding complexes increased during the initial stage of the simulation and gradually reached a relatively stable fluctuation after approximately 40 to 60 ns. Among them, apo C3 reached a stable plateau earlier, whereas the RMSD of apo C5 exhibited noticeable fluctuations in the later stage. In comparison, most ligand-bound complexes displayed relatively stable RMSD profiles, suggesting that ligand binding may modulate the dynamic conformational fluctuations of the protein. Notably, the C3/kaempferol complex showed a more pronounced RMSD fluctuation around 50 to 60 ns, which may indicate a local conformational adjustment or a rearrangement of the binding mode. The RMSD of the ligand relative to the protein backbone was also calculated to assess the positional stability of the ligand within the binding pocket. The results demonstrated that, except for the C3/isorhamnetin, C3/kaempferol, and C5/isorhamnetin complexes, all other ligands maintained stable conformations within their respective protein binding sites during the simulation (Fig. 4B).

### RMSF

The RMSF measures the average positional fluctuations of protein backbone atoms during the simulation, reflecting the flexibility of each amino acid residue. Higher RMSF values typically indicate that the corresponding regions are more flexible or experience greater thermal motion. In general, protein flexibility tends to decrease upon ligand binding, resulting in enhanced structural stability.

Using the apo protein as the baseline reference, C3 exhibited a typical fluctuation pattern during the simulation, with higher flexibility at both termini and relatively stable residues in the central region. Compared with apo C3, the C3/ginkgolide B and C3/isorhamnetin complexes showed a similar overall RMSF distribution pattern. The terminal regions of C3 exhibited relatively high fluctuations, whereas the central residues remained largely stable, with RMSF values below 0.3 nm, suggesting strong rigidity within this region. Kaempferol notably increased the RMSF values at both termini of C3, implying that this ligand may enhance the flexibility of the terminal regions through a specific interaction mechanism. By contrast, quercetin showed an opposite effect, markedly reducing the flexibility of the terminal regions of C3, indicating its stabilizing influence on the protein structure. For complement C5, the apo protein also exhibited relatively higher RMSF values at both terminal regions. Compared with apo C5, the C5/isorhamnetin complex showed markedly increased RMSF values in the terminal and adjacent middle regions, suggesting that isorhamnetin may enhance the conformational flexibility of these regions. In contrast, the overall RMSF distributions of the C5/kaempferol and C5/quercetin complexes were largely comparable to that of the apo protein, with only moderate fluctuations observed in several local regions (Fig. 4D).

### Rg

The Rg represents the mass-weighted root mean square distance of protein atoms from their center of mass and is used to evaluate the overall size and conformation of a protein. During MD simulations, variations in Rg can reflect large-scale conformational transitions such as protein folding or unfolding.

Our analysis indicated that the C3/ginkgolide B, C3/isorhamnetin, and C3/quercetin systems exhibited similar convergence behavior to apo C3, whereas the C3/kaempferol complex showed a marked conformational expansion after 60 ns. This observation corresponded with the finding that kaempferol was unable to maintain a stable position within the binding pocket of C3 beyond 60 ns. For the C5 systems, the Rg profiles of C5/kaempferol and C5/quercetin were generally comparable to that of apo C5, with the quercetin complex showing slightly lower Rg values, suggesting a relatively more compact conformation. In contrast, the C5/isorhamnetin system displayed greater fluctuations in Rg values throughout the simulation, suggesting that the unstable affinity between isorhamnetin and C5 may have contributed to this phenomenon (Fig. 4C).

### Hydrogen Bond Distribution

Because hydrogen bonds are one of the major forces contributing to the stable association between proteins and small-molecule ligands, the number of hydrogen bonds formed between each protein and its corresponding ligand was monitored at every time point during the simulation.

The results showed that ginkgolide B and quercetin formed relatively stable and abundant hydrogen bonds with C3, suggesting that hydrogen bonding may be a key factor responsible for their high binding affinity to the protein. In contrast, kaempferol and isorhamnetin formed fewer and less persistent hydrogen bonds with C3, indicating that hydrogen bonding plays a relatively minor role in their interactions with the protein and that other noncovalent interactions may contribute to ligand stabilization. Similarly, both kaempferol and quercetin formed more stable hydrogen bonds with C5, consistent with the RMSD results of the ligands relative to the protein backbone. In comparison, isorhamnetin formed fewer and less stable hydrogen bonds with C5, suggesting a limited contribution of hydrogen bonding to its interaction with the protein (Fig. 4E).

### MM-PBSA Binding Free Energy and Per-Residue Energy Decomposition

The MM-PBSA method was employed to calculate the binding free energies between proteins and ligands using the last 20 ns of the simulation trajectories. This approach provides a more reliable estimation of binding affinity than docking calculations, as it accounts for the dynamic behavior of molecular systems.

The calculated binding free energies for the C3/ginkgolide B, C3/isorhamnetin, C3/kaempferol, C3/quercetin, C5/isorhamnetin, C5/kaempferol, and C5/quercetin complexes were −18.03 ± 3.45 kcal/mol, −4.42 ± 6.23 kcal/mol, −8.97 ± 1.69 kcal/mol, −16.40 ± 3.28 kcal/mol, −9.46 ± 5.85 kcal/mol, −12.84 ±1.54 kcal/mol, and −19.70 ± 3.68 kcal/mol, respectively. These binding energies were primarily contributed by van der Waals and electrostatic interaction energies. The results were consistent with the RMSD data of ligands relative to the protein backbone, indicating that complexes with lower binding free energies exhibited stronger ligand affinity and greater conformational stability.

Subsequently, a per-residue energy decomposition analysis was performed within a 5 Å radius around each ligand to identify the residues contributing most to the total binding energy. For C3, residues ASP1435, GLU1433, LYS1001, and LYS1436 contributed significantly to the binding of quercetin, kaempferol, and isorhamnetin, while ARG1441, ARG937, ASP1404, ASP1435, and ASP1440 were the major contributors to the binding of ginkgolide B. The difference in the binding patterns may be attributed to the distinct molecular structure of ginkgolide B compared with the other three flavonoids. For C5, residues ASP1457, GLU837, and GLU932 made the greatest energetic contributions to the binding of quercetin, kaempferol, and isorhamnetin (Fig. 4F).

## Discussion

This study provides the first systematic molecular-level investigation of the potential mechanisms of EGb 761 in the treatment of dry AMD. By integrating bioinformatics analysis with MD simulations, we found that most ligand–protein complexes exhibited reduced conformational fluctuations compared with the unbound proteins, suggesting that ligand binding may partially restrict excessive structural dynamics and thereby enhance the overall conformational stability of the complexes^28^; further analysis showed that the major active constituents of EGb 761, including quercetin, kaempferol, isorhamnetin, and ginkgolide B, exhibited stable binding to the key complement proteins C3 and C5. Among these, the C3/quercetin, C3/ginkgolide B, C5/kaempferol, and C5/quercetin complexes demonstrated the greatest thermodynamic stability during MD simulations.

The MM-PBSA analysis revealed that van der Waals and electrostatic forces were the primary contributors to the binding affinity between these ligands and complement proteins. Notably, quercetin and ginkgolide B displayed the lowest binding free energies, indicating the strongest affinities. Furthermore, per-residue energy decomposition identified several critical amino acid residues within the C3b and C5b domains, including ASP1435, GLU1433, LYS1001, and ASP1457, which contributed substantially to ligand binding. Further analysis revealed that the key interacting residues within the C3 binding pocket varied among different EGb 761 constituents. In this study, quercetin showed prominent energy contributions with residues such as ASP1435, GLU1433, and LYS1001, whereas ginkgolide B interacted more strongly with residues including ARG1441, ARG937, and ASP1404. This distinction suggests that quercetin and ginkgolide B may occupy partially distinct microenvironments within the C3 binding pocket, potentially producing cooperative regulatory effects at the same target site.

These findings suggest that EGb 761 may attenuate excessive complement activation through direct interaction with key residues in the complement cascade, providing new molecular-level evidence for its potential therapeutic value in dry AMD.

This study also underscores the pivotal role of the complement alternative pathway in the pathogenesis and progression of dry AMD, consistent with previous findings.^29^ Advances in high-throughput sequencing have clarified the causal relationship between CFH gene variants and AMD susceptibility.^30^ In 2023, the US Food and Drug Administration approved the complement inhibitors pegcetacoplan (targeting C3) and avacincaptad pegol (targeting C5) for the treatment of geographic atrophy (GA),^31^^,^^32^ highlighting the therapeutic importance of complement modulation. In fact, the alternative pathway is actively involved throughout the entire course of AMD, from early to late stages. In early AMD, RPE cells are subjected to oxidative stress and blue light damage, leading to lipofuscin accumulation, decreased RPE function, and reduced CFH expression. These alterations result in elevated levels of C3, CFB, and the terminal complement complex (C5b–9), indicating aberrant activation of the complement pathway. The subsequent formation of the membrane attack complex (MAC) further induces inflammatory responses and RPE apoptosis. As the disease progresses, RPE cell death and Bruch's membrane disruption become more pronounced, while persistent complement activation and elevated C5b–9 levels lead to photoreceptor loss and retinal degeneration.^33^^,^^34^ When one eye progresses to late-stage AMD, marked neurodegenerative changes have typically occurred. Therefore, early intervention aimed at delaying disease progression is critical.^8^^,^^35^ However, pegcetacoplan and avacincaptad pegol are currently approved only for late-stage GA, and effective therapeutic strategies for the early stages of the disease remain limited. Given that complement cascade activation persists throughout the entire course of AMD from early to advanced stages, the mechanism identified in this study, whereby EGb 761 may modulate the complement cascade through interactions with C3 and C5, suggests that it can provide a treatment option for early AMD.^36^^,^^37^

*Ginkgo biloba*, known as a “living fossil” in traditional Chinese medicine, has been used in China for over 5000 years. In 1965, Germany developed the first pharmaceutical preparation of *G.* *biloba* extract, and in 1974, the first commercial formulation was approved for human use in France under the name EGb 761.^38^ Historically, the pharmacologic benefits of EGb 761 have been attributed mainly to its antioxidant, vasodilatory, and antiplatelet activating properties. To survive in terrestrial environments, higher plants evolved structural and chemical defense systems to protect cells from oxidative injury, and antioxidant capacity remains a key adaptive trait. As most natural metabolites cannot be fully reproduced by chemical synthesis, they retain significant biological advantages.^39^ Distinct from its classical antioxidant and circulatory effects, the present study revealed that the active components of EGb 761, particularly ginkgolide B, kaempferol, and quercetin, could directly bind to complement proteins C3 and C5, modulate complement activation, and stabilize protein conformations. These findings provide a new mechanistic basis for understanding EGb 761 and support its potential as a natural complement-modulating agent.

This study, primarily based on bioinformatics and molecular dynamics simulations, represents an early stage of molecular mechanism discovery but provides valuable insights for future translational research. The identified binding targets, complement components C3 and C5, coincide with those of currently approved complement inhibitors, indicating the direct clinical relevance of these computational findings. Moreover, EGb 761 has demonstrated good safety and tolerability in both neurodegenerative and ophthalmic applications, offering a realistic foundation for potential clinical translation. Clinically, as a multicomponent botanical formulation, EGb 761 exhibits antioxidant, anti-inflammatory, and neuroprotective properties through multiple pathways.^40^^–^^42^ Its potential complement-modulating activity may help overcome the limitations of existing single-target therapies.

This study has certain limitations. The molecular dynamics simulations were performed under idealized conditions without accounting for protein posttranslational modifications or the complexity of the in vivo physiological environment. Future in vitro and in vivo experiments are warranted to validate the computational predictions and further elucidate the molecular basis of drug–target interactions, which will facilitate the development of early, noninvasive therapeutic strategies for dry AMD.

In conclusion, our study systematically demonstrated that EGb 761 may exert protective effects in dry AMD by stably binding to complement proteins C3 and C5 and inhibiting excessive complement activation. These findings provide theoretical support for the therapeutic potential of EGb 761 in dry AMD and offer new insights for developing complement-targeted drugs derived from natural products.
